# In1-ghrelin splicing variant is overexpressed in pituitary adenomas and increases their aggressive features

**DOI:** 10.1038/srep08714

**Published:** 2015-03-04

**Authors:** Alejandro Ibáñez-Costa, Manuel D. Gahete, Esther Rivero-Cortés, David Rincón-Fernández, Richard Nelson, Manuel Beltrán, Andrés de la Riva, Miguel A. Japón, Eva Venegas-Moreno, Ma Ángeles Gálvez, Juan A. García-Arnés, Alfonso Soto-Moreno, Jennifer Morgan, Natia Tsomaia, Michael D. Culler, Carlos Dieguez, Justo P. Castaño, Raúl M. Luque

**Affiliations:** 1 Department of Cell Biology, Physiology and Immunology, University of Cordoba, Instituto Maimónides de Investigación Biomédica de Córdoba (IMIBIC), Hospital Universitario Reina Sofia; CIBER Fisiopatología de la Obesidad y Nutrición; and Campus de Excelencia Internacional Agroalimentario (ceiA3), 14014 Córdoba, Spain; 2 IPSEN Bioscience, Cambridge, 02142 Massachusetts, USA; 3 Department of Pathology, Puerta del Mar University Hospital, Cádiz; 4 Service of Neurosurgery, Hospital Universitario Reina Sofia, 14004 Córdoba, Spain; 5 Department of Pathology, Hospital Universitario Virgen del Rocío, 41013 Seville, Spain; 6 Metabolism and Nutrition Unit, Hospital Universitario Virgen del Rocío, Instituto de Biomedicina de Sevilla (IBIS), 41013 Seville, Spain; 7 Service of Endocrinology and Nutrition, Hospital Universitario Reina Sofia, and Instituto Maimónides de Investigación Biomédica de Córdoba, 14004 Córdoba, Spain; 8 Department of Endocrinology and Nutrition, Carlos Haya Hospital, 29010 Málaga, Spain; 9 Department of Physiology, University of Santiago de Compostela, and CIBER Fisiopatología de la Obesidad y Nutrición, 15782 Santiago de Compostela, Spain

## Abstract

Pituitary adenomas comprise a heterogeneous subset of pathologies causing serious comorbidities, which would benefit from identification of novel, common molecular/cellular biomarkers and therapeutic targets. The ghrelin system has been linked to development of certain endocrine-related cancers. Systematic analysis of the presence and functional implications of some components of the ghrelin system, including native ghrelin, receptors and the recently discovered splicing variant In1-ghrelin, in human normal pituitaries (n = 11) and pituitary adenomas (n = 169) revealed that expression pattern of ghrelin system suffers a clear alteration in pituitary adenomasas comparedwith normal pituitary, where In1-ghrelin is markedly overexpressed. Interestingly, in cultured pituitary adenoma cells In1-ghrelin treatment (acylated peptides at 100 nM; 24–72 h) increased GH and ACTH secretion, Ca^2+^ and ERK1/2 signaling and cell viability, whereas In1-ghrelin silencing (using a specific siRNA; 100 nM) reduced cell viability. These results indicate that an alteration of the ghrelin system, specially its In1-ghrelin variant, could contribute to pathogenesis of different pituitary adenomas types, and suggest that this variant and its related ghrelin system could provide new tools to identify novel, more general diagnostic, prognostic and potential therapeutic targets in pituitary tumors.

*Ghrelin* gene (*GHRL*) products and associated receptors, proteins, and enzymes are emerging as an intricate and pleiotropic regulatory system involved in a plethora of physiological and pathological functions, including hormonal secretions and tumor development and progression^1^,^2^. *GHRL* gene encompasses four coding exons^3^ that alternatively combine, through splicing processes, to generate several mature and functional mRNAs, which, after translation, generate prepro-peptides that are further processed by the action of proteolytic enzymes to originate biologically active peptides^4^ (e.g. native ghrelin, obestatin, etc.). Among them, special attention has been dedicated to ghrelin itself, a 28-aa peptide hormone, including its acylated (AG) and unacylated forms, obestatin^5^, and more recently, to their splicing variants^1^,^4^,^6^,^7^,^8^,^9^. Among these splicing variants is the In1-ghrelin variant, which is generated by retention of intron 1 (In1) resulting in an alteration in the amino acids (aa) sequence of the C-terminal portion as compared with native-ghrelin. However, In1-ghrelin variant shares the signal peptide and an initial portion of 13 aa of its peptide sequence with native ghrelin, which includes the first 5-amino acids (aa) that comprises the minimum sequence required for ghrelin acylation by MBOAT4, the enzyme responsible for ghrelin acylation^10^,^11^, and for binding and activation of GHSR-1a^1^,^4^. Therefore, In1-ghrelin variant would encode a different prepro-peptide that conserves the initial aa of native ghrelin but presents a different C-terminal tail, and whose expression has been demonstrated in several human healthy tissues, and has been found to be overexpressed in breast cancer^6^. Moreover, the orthologous counterparts of the human In1-ghrelin variant have also been found in mice (named In2-ghrelin^12^) and in a non-human primate model^6^, which suggest that this new variant might exert an important physiological role that is conserved across mammalian species.

*GHRL* gene-derived transcripts/peptides are produced by the pituitary gland^6^,^13^,^14^, and seem to be involved in the regulation of the normal pituitary secretory pattern^1^,^15^,^16^,^17^. In contrast to the growing number of biologically active ghrelin gene-derived peptides, only a single receptor, transcribed from *GHSR* gene, named GHSR1a, has hitherto been identified as unequivocal endogenous functional binding target for AG, while a physiological function has not been unequivocally ascribed to its shorter, truncated splicing isoform GHS-R1b^18^. On the other hand, the receptor(s) mediating the actions of unacylated-ghrelin, obestatin, In1-ghrelin and other splicing variants remain elusive, if not controversial^18^. The enzyme responsible for ghrelin acylation, MBOAT4, belongs to the superfamily of membrane bound O-acyltransferases, and is commonly referred to as ghrelin-O-acyltransferase (GOAT)^10^,^11^. This enzyme has been found to be expressed in a variety of human and rodent tissues^19^,^20^, including the pituitary, where it has been proposed that locally produced GOAT might possibly be active to convertlocally produced or circulating non-acylated forms of proghrelin or proIn1-ghrelin to their acylated forms to mediate tissue-specific effects^20^.

The first evidence indicating that ghrelin system could be involved in tumor development and/or progression was the finding that GHSR1a was expressed in normal and tumoral pituitaries^21^. Thereafter, ghrelin was also found in various types of pituitary tumors^13^,^22^,^23^, thus suggesting a complex autocrine/paracrine role of the ghrelin system in pituitary pathogenesis. In fact, ghrelin, GHSR1a and the truncated GHSR1b have been found to be expressed in a wide variety of endocrine-related tumors, including pituitary adenomas, neuroendocrine tumors and breast and prostate tumors^6^,^13^,^21^,^24^,^25^. Additionally, MBOAT4 and In1-ghrelin variant expression has been observed in breast^6^,^26^ and prostate^27^,^28^,^29^ cancers but their presence in pituitary adenomas is still to be determined.

Although some of the components of the ghrelin system seems to exert autocrine/paracrine regulatory actions and could thus hold potential as a diagnostic, prognostic or therapeutic target in several tumoral pathologies, including pituitary adenomas, its exact role in tumor development and progression is still uncertain. Moreover, the presence of MBOAT4 and In1-ghrelin has not yet been determined in pituitary adenomas. Therefore, the aim of this study was to systematically analyze, for the first time, the side-by-side presence of different components of the ghrelin system: native ghrelin, In1-ghrelin variant, MBOAT4 enzyme, GHSR1a and GHSR1b, in normal pituitaries and in all major types of human pituitary tumors. In addition, we used pituitary adenoma primary cell cultures and a mouse corticotropinoma cell line (AtT-20) to compare the direct effects of native ghrelin and In1/In2-ghrelin variant administration on selected functional parameters to better define the pathophysiological significance of this regulatory system in pituitary tumors.

## Results

### Ghrelin system is altered in pituitary adenomas compared to normal pituitary

**Figure 1:**
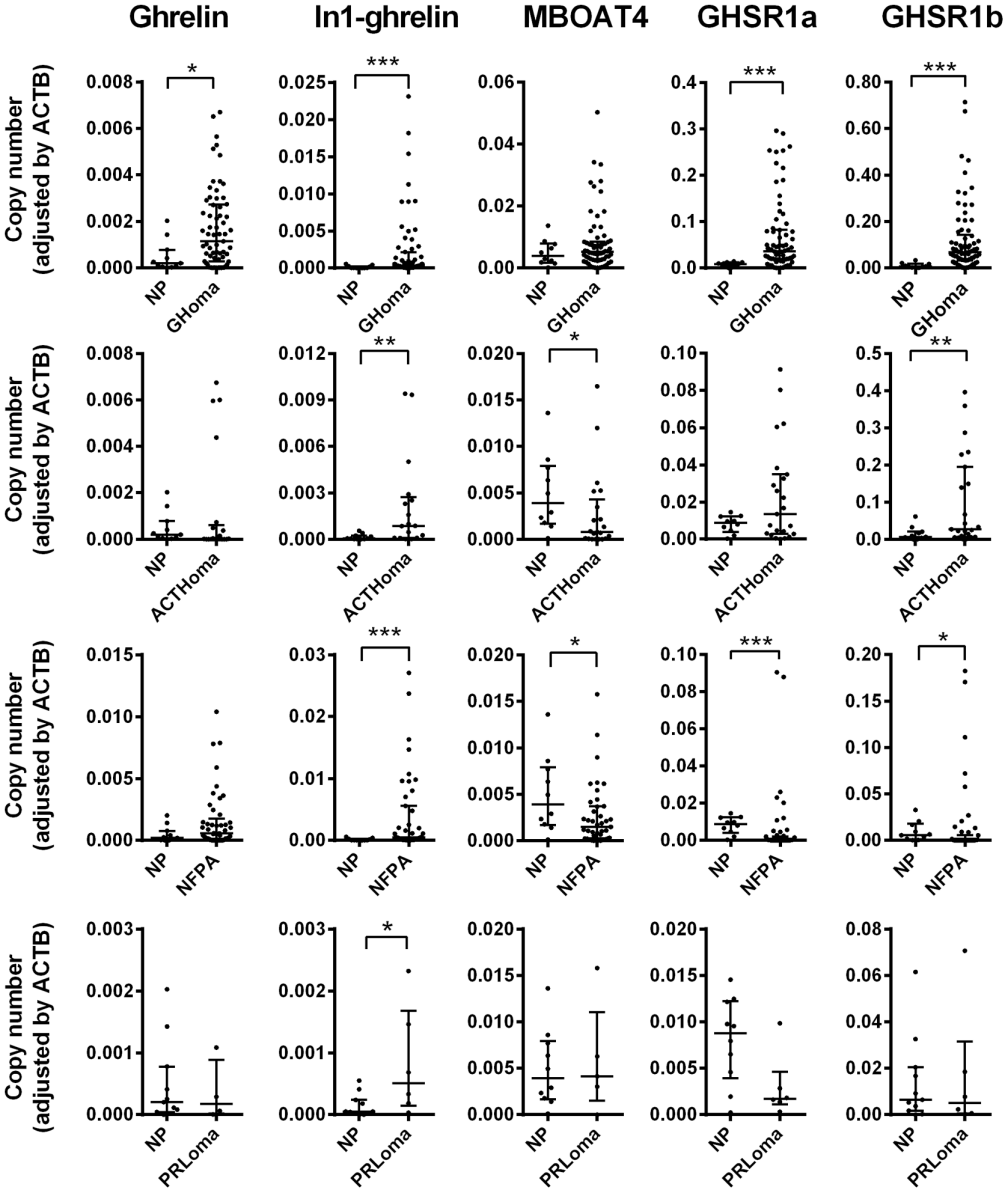
Expression profile of ghrelin system components in normal and tumoral pituitaries. The expression of ghrelin system components (ghrelin, In1-ghrelin variant, MBOAT4, GHSR1a and GHSR1b) was determined by qPCR in a battery of 169 pituitary adenomas (including 76 GH-omas, 29 ACTH-omas, 57 NFPAs and 7 PRL-omas) and compared to the expression levels found in 11 normal pituitaries (NPs). Data represent median ± interquartile range of absolute expression levels (copy number) of each transcript adjusted by the expression levels of a control gene (ACTB). Asterisks (*, p < 0.05; **, p < 0.01 and ***, p < 0.001) indicate data that differ from normal pituitary expression by Mann-Whitney U test.

All components of the ghrelin system examined (native ghrelin, In1-ghrelin variant, GHSR1a, GHSR1b and MBOAT4 enzyme) were expressed in normal pituitaries (Figure 1 and Supplemental Figure-1). Expression of MBOAT4 and that of ghrelin receptors were also found at appreciable levels in normal pituitaries (Figure 1). Interestingly, the expression pattern of ghrelin system components was markedly altered in pituitary adenomas compared with normal pituitaries. Specifically, median expression levels of native ghrelin were significantly elevated in GH-omas, while no significant changes were observed in ACTH-omas, NFPAs or PRL-omas compared with normal pituitaries (Figure 1 and Supplemental Figure-1). In contrast, the expression of In1-ghrelin was significantly elevated in all types of pituitary adenomas analyzed (Figure 1 and Supplemental Figure-1), suggesting a potential common relevant role in pituitary tumors pathogenesis. Expression of MBOAT4 was slightly, but significantly, reduced in ACTH-omas and NFPAs compared to normal pituitaries, while no significant changes were observed in GH-omas and PRL-omas (Figure 1 and Supplemental Figure-1). In the case of ghrelin receptors, a pronounced overexpression of GHSR1a and GHSR1b was observed in GH-omas and, similarly, an elevation in the expression of both receptors was also observed in ACTH-omas, which was only statistically significant for GHSR1b. In contrast, median expression levels of both receptors were reduced in NFPAs, and no changes were observed in PRL-omas (Figure 1 and Supplemental Figure-1). Although caution should be exerted when comparing normal pituitary tissues (which contain a mixture of pituitary cell subtypes) and pituitary adenomas (though to be mono/oligoclonal in origin), it should be noted that the careful use of this type of comparison has been generally accepted^13^,^30^ as it provides potentially valuable and informative insights, and may help to set the stage for future investigations to elucidate the putative physiological role(s) for the ghrelin system in normal pituitaries and pituitary adenomas. In1-ghrelin expression levels correlated with those of MBOAT4 in GH-omas, ACTH-omas, and NFPAs, but not in normal pituitaries (no correlation analysis were performed in PRL-omas due to insufficient number of samples), which may suggest that In1-ghrelin could be functionally linked with MBOAT4 in pituitary adenomas. Expression levels of ghrelin and MBOAT4 correlated in GH-omas and NFPAs but not in ACTH-omas and normal pituitaries (Supplemental Table-2). Inasmuch as In1-ghrelin expression was consistently elevated in all pituitary adenomas, and its expression correlated with that of MBOAT4 in pituitary adenomas (and not in normal pituitaries), we considered of foremost interest to explore the putative role of this novel ghrelin gene variant in the pathophysiology of pituitary tumors.

### In1-ghrelin peptides induce intracellular signaling activation in pituitary adenoma cells

**Figure 2:**
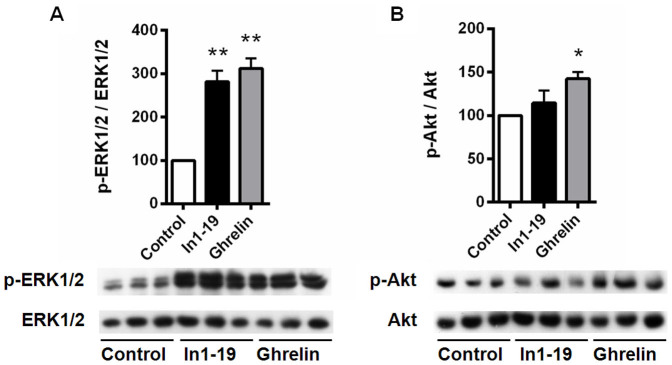
Basal ghrelin- and In1-19-induced phosphorylation of ERK1/2 and Akt in somatotropinomas. Representative Western Blots and quantification of levels of p-ERK1/2/total ERK1/2 and p-Akt/total Akt in response to ghrelin and In1-19 (100 nM) on GHomas (n = 4). Data are expressed as percent of vehicle-treated controls (set at 100%) within experiment. Values represent the mean ± S.E.M. Asterisks (* p < 0.05, ** p < 0.01) indicate significant differences between vehicle- and peptide-treatments.

To test the capacity of In1-ghrelin peptides to induce functional responses in pituitary tumor cells, we measured the kinetics of free cytosolic calcium concentration ([Ca^2+^]_i_) in single cells derived from GH-omas, ACTH-omas, NFPAs and PRL-omas, as well as the levels of phosphorylation of two signaling pathways (MAPK and Akt; p-ERK1/2 or p-Akt, respectively) in GH-omas in response to treatment with acylated In1-ghrelin peptides as compared with that of AG. Calcium is a relevant second messenger well known for its involvement on pituitary cell physiology, where it triggers hormone secretion and has thus been widely used to evaluate pituitary cell function^23^. Therefore, two putative In1-ghrelin derived peptides (In1-19 and In1-40) were chemically synthesized and employed, in parallel with AG. AG induced [Ca^2+^]_i_ changes in the majority of cells derived from all GH-omas and ACTH-omas tested (Table 2). Particularly, 74% of the cells from GH-omas and ACTH-omas responded to AG doubling the [Ca^2+^]_i_ levels. Remarkably, both In1-ghrelin peptides induced similar responses in most GH-oma and ACTH-oma cells, with In1-19, in general terms, being more efficient than In1-40. In all cases, the increase in [Ca^2+^]_i_ levels were slightly lower than those elicited by AG (Table 2). In NFPA, AG and In1-ghrelin peptides elicited [Ca^2+^]_i_ responses in approximately 50% of the adenoma cultures, with lower proportion of responsive cells (29%, 18% and 10%, respectively) and lower maximal responses than those observed in GH- and ACTH-omas. Finally, PRL-oma cells showed a very modest response to ghrelin system peptides with less than 20% of cells increasing [Ca^2+^]_i_ levels in response to AG and less that 10% of cells responding to In1-ghrelin peptides in only a subset of the PRL-omas. In1-ghrelin peptides exhibited a slightly lower potency than AG in all parameters tested in NFPAs and PRL-omas (Table 2). Altogether, these results demonstrate that AG and In1-ghrelin peptides impact Ca^2+^ signaling in pituitary adenoma cell cultures. To test the possibility that In1-ghrelin peptides could bind/activate GHSRs, CHO-K1 cells stably transfected with either GHSR1a or GHSR1b were employed to analyze [Ca^2+^]_i_ levels in response to In1-ghrelin peptides. Both, In1-19 and In1-40 induced [Ca^2+^]_i_ changes in GHSR1a-transfected cells, with similar EC_50_ to that exhibited by ghrelin (Supplemental Table-3); while GHSR1b-transfected cells did not respond to any of the peptides tested (EC_50_ > 3000); therefore suggesting that In1-ghrelin peptides can act, at least in part, through GHSR1a. Nevertheless, responses to AG^18^ and In1-ghrelin (data not shown) have been found in GHSR-lacking cells (e.g. prostate cell lines), suggesting the existence of additional receptors of the ghrelin system. Interestingly, treatment with both AG and acylated In1-19 induced a clear increase on p-ERK1/2 in somatotropinomas as compared to vehicle treated controls (Figure 2A), whereas only AG, but not In1-19, was able to increase p-Akt levels (Figure 2B).

### In1-ghrelin peptides induce hormone secretion from pituitary adenoma cells

**Figure 3:**
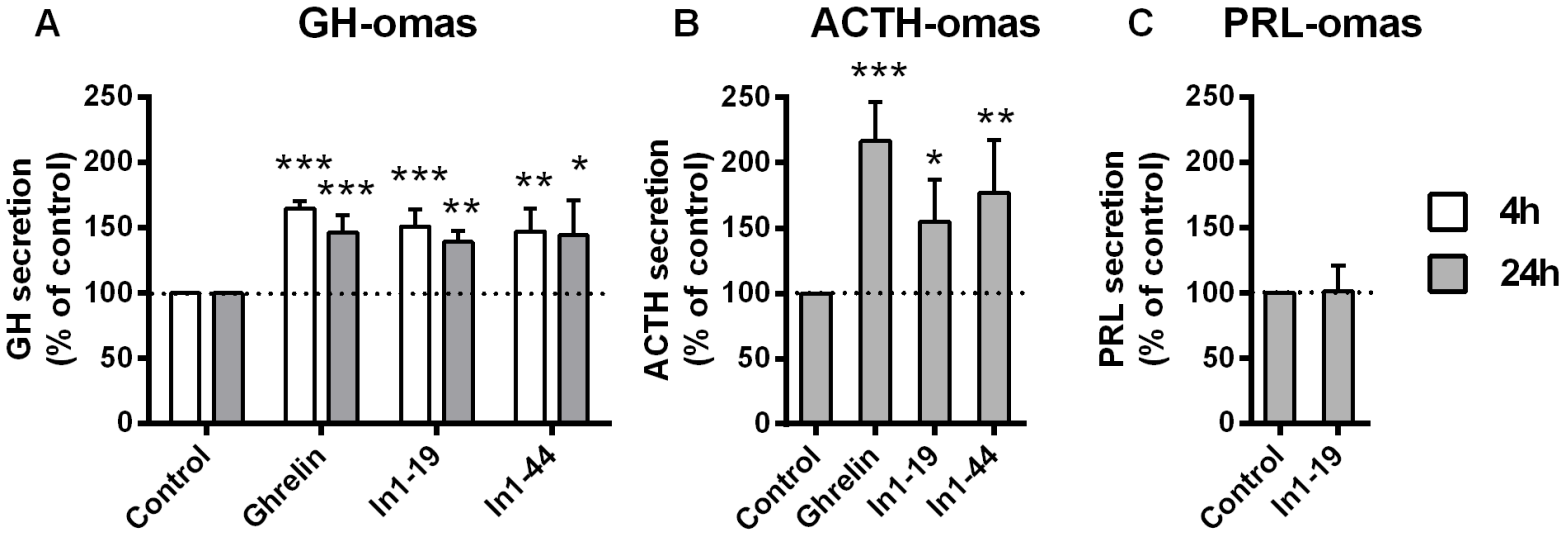
Hormone secretion in response to ghrelin and In1-ghrelin in tumoral pituitaries. Effect of 4- and/or 24-h treatment of ghrelin (100 nM) and/or In1-ghrelin derived peptides (In1-19 and In1-40; 100 nM) on GH, ACTH and PRL release from human GH-omas (A; n = 3–7), ACTH-omas (B; n = 3–9), and PRL-omas (C; n = 2) primary cell cultures, respectively, determined by commercial ELISA kits. Data are expressed as percent of vehicle-treated controls (set at 100%) within experiment. Values represent the mean ± S.E.M. Asterisks (* p < 0.05, ** p < 0.01; *** p < 0.001) indicate significant differences between vehicle- and peptide-treatments.

**Figure 4:**
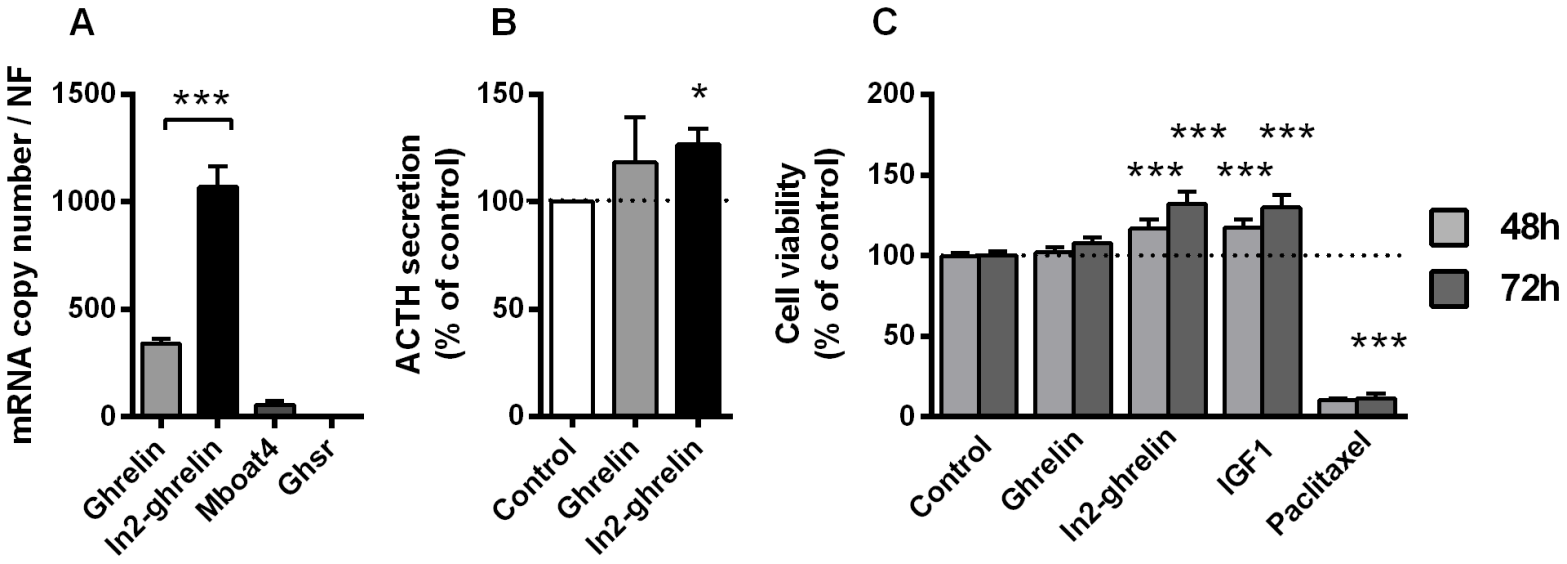
Ghrelin and In2-ghrelin variant in AtT20 cell line. (A) Expression profile of the ghrelin system (Ghrelin, In2-ghrelin variant, Mboat4 enzyme and Ghsr) in AtT-20 cell line obtained by qPCR. Data represent absolute mRNA copy number adjusted by a normalization factor (NF) calculated from the expression levels of three control genes (Actb, Ppia and Hprt); (B) ACTH secretion in response to 4 h ghrelin and In2-ghrelin variant treatment; (C) Cell viability in response to ghrelin and In2; IGF-1 and Paclitaxel were used as positive and negative controls, respectively. Values represent the mean ± S.E.M of 3–5 independent experiments. Asterisks (* p < 0.05, *** p < 0.001) indicate data that significantly differ from controls.

To confirm that the responsiveness of pituitary adenoma cells to In1-ghrelin peptides was translated into further functional outcomes, *in vitro* hormone secretion was assessed in a subset of tumors in response to In1-ghrelin peptides, and, when cells were available, also to AG. AG and In1-ghrelin peptides, similarly and significantly, increased GH secretion in GH-omas (4 h and 24 h treatments; Figure 3A). Likewise, AG and In1-ghrelin peptides significantly increased ACTH secretion in ACTH-omas with similar efficacy, after 24 h treatment (Figure 3B). In contrast, In1-19 was not able to alter PRL secretion in the samples tested (n = 2) after 24 h treatment (Figure 3C). Unfortunately, we could not investigate the effect of AG or In1-40 peptide in PRL-omas due to the limited source of preparations and of the number of cells obtained after dispersion. Finally, the results obtained in human ACTH-omas were further supported by the use of a widely accepted cellular model for ACTH-producing adenomas, the AtT-20 cell line. Specifically, AtT-20 cell line expressed higher levels of In2-ghrelin [the murine counterpart of In1-ghrelin^12^] compared with native ghrelin (Figure 4A), a situation similar to that found in human ACTH-omas where In1-ghrelin expression was higher than native ghrelin expression (p < 0.01; data not shown). Treatment with AG did not significantly alter basal ACTH release in AtT-20 cell line (which might be explained by the lack of GHSR expression; Figure 4A and 4B), while treatment with In2-ghrelin peptide significantly increased basal ACTH release which further support the existence of additional receptors of the ghrelin system.

### In1-ghrelin enhances cell viability in pituitary adenoma cells

**Figure 5:**
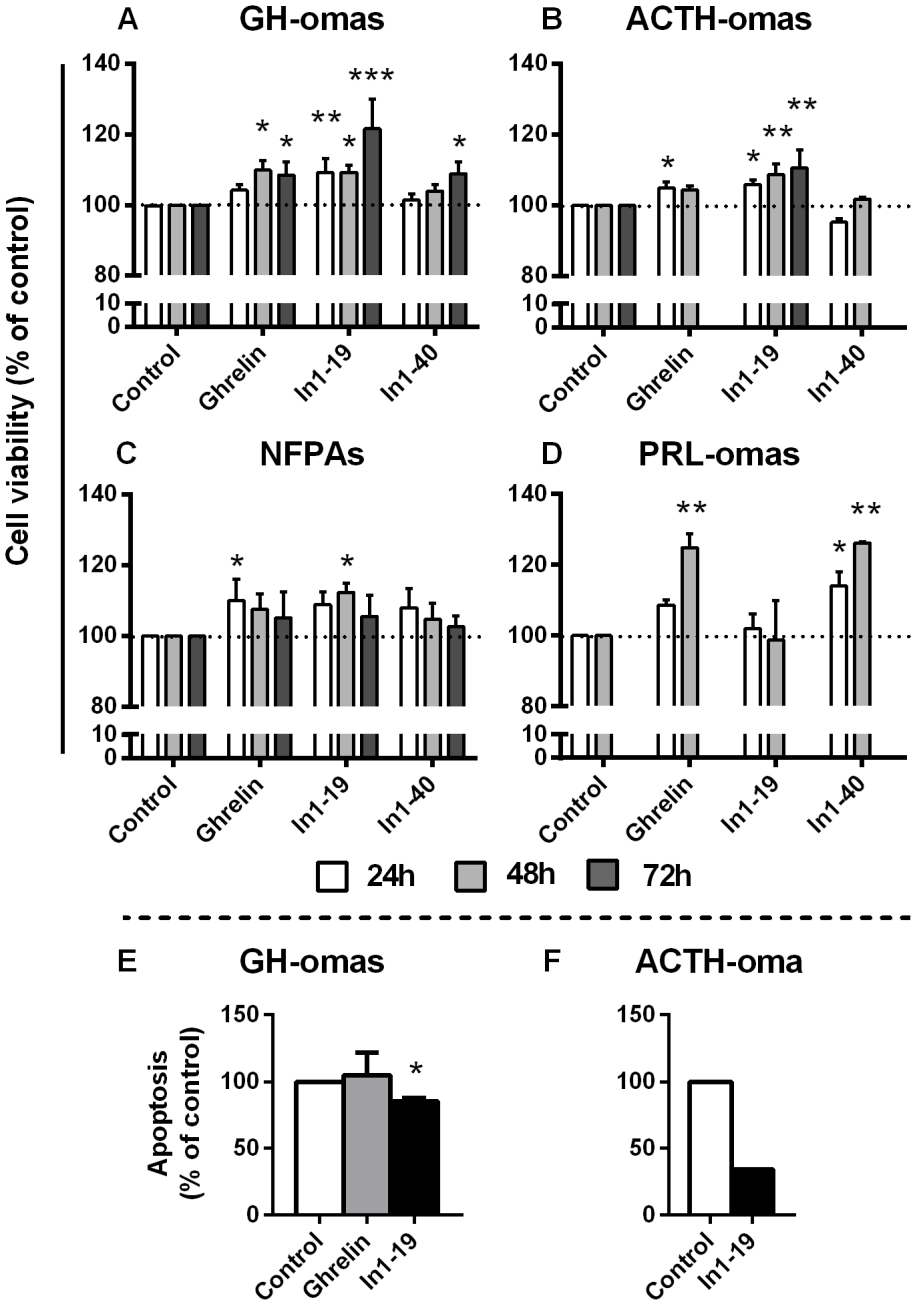
Cell viability and apoptosis in response to In1-ghrelin and/or ghrelin in tumoral pituitaries. Effect of 24-, 48- and/or 72-h treatment of acylated ghrelin (100 nM) and/or acylated In1-ghrelin derived peptides (In1-19 and In1-40; 100 nM) on cell viability in human GH-omas (A; n = 5–17), ACTH-omas (B; n = 2–8), NFPAs (C; n = 3–15), and PRL-omas (D; n = 2–3) primary cell cultures, determined by alamar-blue reduction. Effect of 12–14 h treatment with acylated ghrelin and In1-19 (100 nM) on apoptotic rate in human GH-omas (E; n = 3) and ACTH-oma (F; n = 1), determined by Annexin V/Propidium Iodide staining. Data are expressed as percent of vehicle-treated controls (set at 100%) within experiment. Values represent the mean ± S.E.M. Asterisks (* p < 0.05, ** p < 0.01; *** p < 0.001) indicate significant differences between vehicle- and peptide-treatments.

Administration of AG and In1-ghrelin peptides increased cell viability *in vitro* in all pituitary adenoma subtypes (Figure 5). In particular, cell viability was significantly increased in GH-oma cell cultures after 48 and 72 h treatment in response to AG; after 24, 48 and 72 h treatment with In1-19 and after 72 h treatment with In1-40, with In1-19 apparently being more effective than AG and In1-40 at 72 h (Figure 5A). In ACTH-omas, AG increased cell viability at 24 h; while, In1-19 increased cell viability at 24, 48 and 72 h, and In1-40 did not exert any effect (Figure 5B). Consistent with an effect of In1-ghrelin on ACTH-oma cell viability, In2-ghrelin peptide stimulated cell viability on AtT-20 cell line, while AG did not alter cell viability in these cells (Figure 4C). Furthermore, AG and In1-ghrelin peptides exhibited modest but consistent increments in cell viability in NFPAs, reaching significant levels at 24 h for AG and at 48 h for In1-19 (treatment with In1-40 did not alter cell viability in NFPA-cells) (Figure 5C). Finally, in PRL-oma cultures, AG and In1-40 but not In1-19 elicited significant increases on cell viability (Figure 5D).

### In1-ghrelin decreases apoptotic rate in GH-omas cells

Administration of AG did not alter apoptotic rate as compared to vehicle treated controls in GH-oma cell cultures however; treatment with acylated In1-19 significantly reduced apoptosis in GH-omas (n = 3; Figure 5E) and in one ACTH-oma (n = 1; Figure 5F).

### Effect of overexpression and silencing of In1-ghrelin on in vitro cell viability

**Figure 6:**
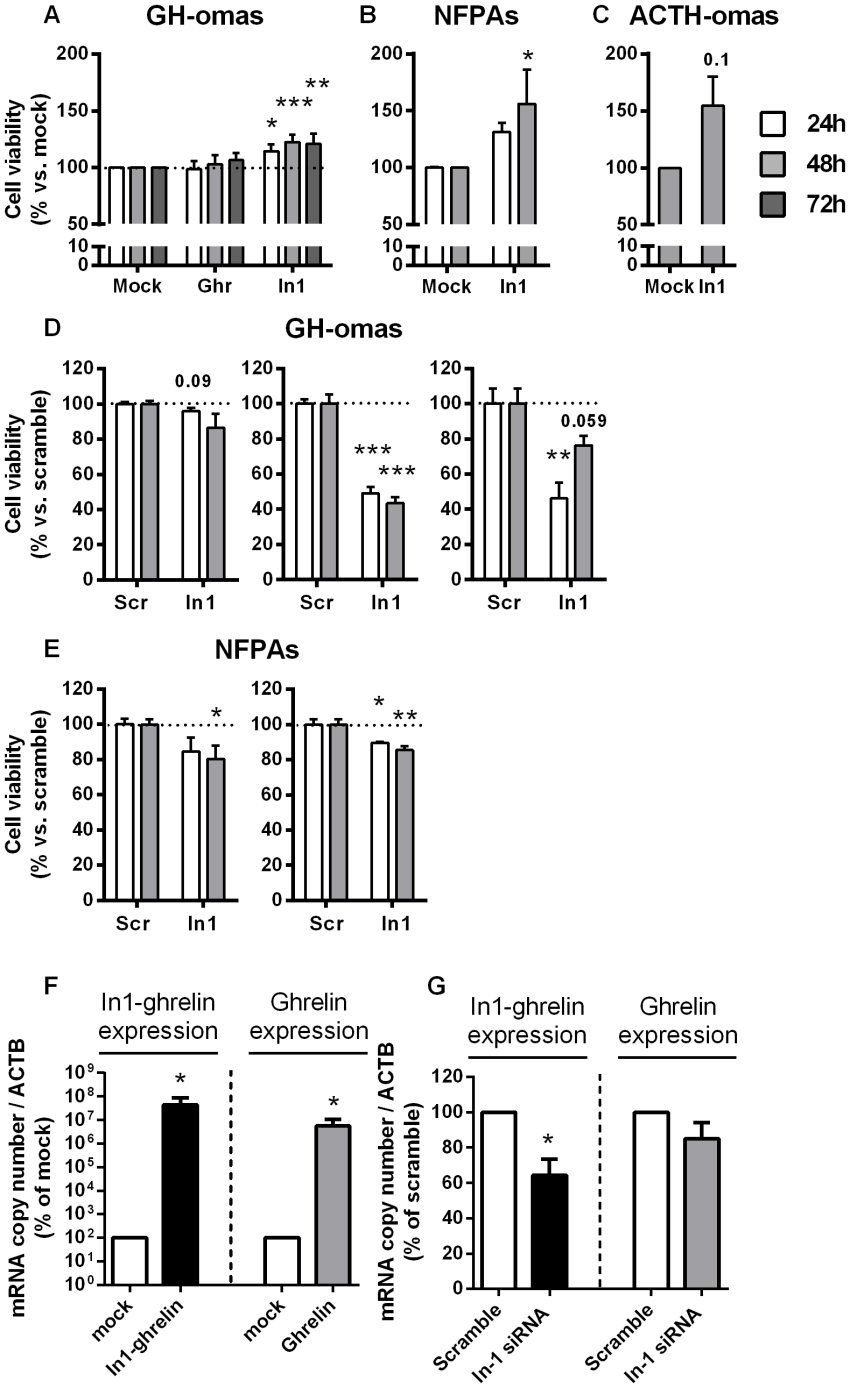
Cell viability in response to In1-ghrelin overexpression and silencing in tumoral pituitaries. Effect of 24-, 48- and/or 72-h overexpression of ghrelin (Ghr) and/or In1-ghrelin (In1) on cell viability in human GH-omas (A; n = 4–11), NFPAs (B; n = 2–3) and ACTH-omas (C; n = 3) primary cell cultures, determined by alamar-blue reduction. Overexpression was induced by transfection with specific expression vectors containing the appropriate CDS of each variant. Effect of 24- and 48-h silencing of In1-ghrelin (In1) expression on cell viability of individual human GH-omas (D; n = 2) and NFPAs (E; n = 2) primary cultures, determined by alamar-blue reduction. In1-ghrelin silencing was induced by transfection with specific siRNAs. Representative validation by qPCR of In1-ghrelin (black bar) and ghrelin (grey bar) overexpression (F), and silencing (G) of In1-ghrelin, demonstrating a decrease of In1-ghrelin mRNA expression (black bar) but no significant decrease of ghrelin mRNA expression (grey bar). Data are expressed as percent of control vectors (A, B, C. Mock; set at 100%) and control random siRNA (D, E. Scr: Scramble; set at 100%) within experiment, respectively. Values represent the mean ± S.E.M. Asterisks (* p < 0.05, ** p < 0.01; *** p < 0.001) indicate data that significantly differ from mock or scramble controls.

In1-ghrelin but not native ghrelin overexpression increased cell viability in cultured GH-oma cells compared to controls (mock-transfected cells) (Figure 6A). Likewise, In1-ghrelin overexpression significantly increased cell viability in NFPA cell cultures (Figure 6B). A similar trend was observed in ACTH-oma cell cultures, where In1-ghrelin overexpression tended to increase cell viability (p = 0.10) (Figure 6C). As mentioned above, analysis of additional treatments (i.e. native ghrelin overexpression) and time-points could not be performed due to limited cell preparations and/or number. Nevertheless, we implemented, as key proof of concept, the opposite approximation, namely In1-ghrelin silencing, in a subset of pituitary adenomas in which cultured cells were available. Validation of the siRNA and selection of the appropriate concentration were carried out using commercially-available cell lines that naturally express In1-ghrelin, such as MDA-MB-231 (breast cancer cell line) and LnCAP (prostate cancer cell line) (data not shown). Specifically, In1-ghrelin expression silencing with a specific siRNA reduced cell viability in two independent GH-oma as well as in two NFPA cell cultures compared to control (scramble-transfected cells) (Figure 6D and 6E, respectively).

## Discussion

Pituitary adenomas have been commonly considered a rare tumoral pathology due to an underestimated diagnosis and, consequently, low incidence worldwide. However, autopsy and imaging studies demonstrated that these tumors present an overall estimated prevalence of 16.7%^31^, thus representing the most common intracranial neoplasms, which are often accompanied by serious comorbidities through mass effects and inappropriate secretion of pituitary hormones. Unfortunately, establishment of effective and long-lasting therapeutic strategies has been hampered by the fact that pituitary adenomas comprise an extremely heterogeneous and complex subset of tumoral pathologies, wherein the finding of common and more global molecular and/or cellular targets for their diagnostic, prognostic and/or therapeutic treatment has been elusive.

In this work, we hypothesized that the ghrelin system could play a role in the regulation of pituitary adenomas, based on previous reports indicating that this system exerts relevant actions in the control of pituitary secretions^1^,^16^,^17^, and that some components of this family are altered in certain pituitary adenomas subtypes^1^,^13^,^21^,^22^,^23^. Thus, it seemed reasonable to propose that identification of altered components of this ghrelin system might be useful in the exploration for new tools for the diagnostic, prognostic and/or therapeutic treatment of pituitary adenomas. To date, a comprehensive and systematic analysis of several of the components of the ghrelin system has not been implemented in parallel in an extensive collection of pituitary adenomas. Moreover, the presence and functional role of the novel ghrelin gene variants, as In1-ghrelin variant, has not been studied in pituitary adenomas. Thus, to better define the pattern of alteration of ghrelin system components in pituitary adenomas, we developed a parallel analysis of the expression pattern of native-ghrelin, In1-ghrelin variant, GHSR1a, GHSR1b and MBOAT4 in a battery of 169 human pituitary adenomas, including GH-, ACTH- and PRL-producing adenomas and NFPAs, compared with normal pituitaries. In line with previous reports, we found that these components were expressed in normal pituitaries^6^,^13^,^19^,^20^,^21^,^32^,^33^. Interestingly, the results generated in this study reveal that, compared to normal pituitaries, expression of the ghrelin system is dramatically altered in pituitary adenomas, which was dependent on tumor subtype and the specific component of the ghrelin system analyzed.

Specifically, expression of *GHRL*-gene derived products (ghrelin and In1-ghrelin), GHSR1a and GHSR1b was markedly elevated in GH-omas, wherein MBOAT4 expression was also slightly albeit non-significantly increased, indicating an overall, profound upregulation of ghrelin system in GH-omas. This study reinforces previous data showing higher expression of ghrelin, GHSR1a and GHSR1b in GH-omas compared to normal pituitaries^22^, and extend this information by demonstrating, for the first time, the presence of appreciable levels of MBOAT4, a key element of this family that is required to activate ghrelin for its binding/action via GHSR1a, and would also presumably acylate the In1-ghrelin variant^6^,^13^,^21^, which, in addition, is heavily overexpressed in GH-omas.

In the rest of tumoral types examined, the alteration of the ghrelin system, although apparent, was more complex. Specifically, in ACTH-omas, where ghrelin was previously shown to be downregulated^13^,^22^ and GHSR1a and GHSR1b upregulated^21^,^22^ compared with normal pituitaries, we found similar tendencies, but with no significant reduction in ghrelin expression and elevation in GHSR1a expression, while GHSR1b expression was significantly elevated. Interestingly, we found a clear upregulation of In1-ghrelin and a reduction in MBOAT4 expression levels in ACTH-omas compared to normal pituitaries. In fact, comparison of the two ghrelin gene derived transcripts expression levels in ACTH-omas (and also in AtT-20 cell line) revealed that the expression of In1-variant was significantly higher than that of native ghrelin, which further supports a relevant role of this variant in ACTH-omas. Conversely, PRL-omas did not exhibit altered ghrelin expression levels, as previously reported^21^,^22^, and neither was GHSR1a and GHSR1b expression altered, which differs with the single report of upregulated ghrelin receptor in PRL-omas^21^. Remarkably, In1-ghrelin expression was also found to be upregulated in PRL-oma samples, while MBOAT4 expression was not altered compared to normal pituitaries. Finally, NFPA samples exhibited a high heterogeneity, probably related to their intrinsically diverse nature, showing a slight elevation in ghrelin expression, which is consistent with previous reports^13^,^22^, and a reduction in GHSR1a and GHSR1b expression levels. Notably, these receptors exhibited a remarkably elevated expression in some NFPA samples, which could be in agreement with previous reports showing an elevation in ghrelin receptor expression in NFPAs^13^,^21^,^22^. In addition, MBOAT4 expression levels were slightly downregulated in NFPAs and, similar to that found in all the pituitary adenomas included in this study, we found that In1-ghrelin was significantly overexpressed in NFPAs.

Taken together, our data confirm earlier studies supporting the existence of a profound alteration of some components of the ghrelin system in the most predominant pituitary adenoma subtypes^13^,^21^,^22^, and extend previous data by demonstrating the expression of In1-ghrelin and MBOAT4 in pituitary adenomas, which further supports the notion that an autocrine/paracrine functional loop involving the ghrelin system can be in place in the pituitary, and may contribute to the (patho)physiological control of the gland.

**Figure 7:**
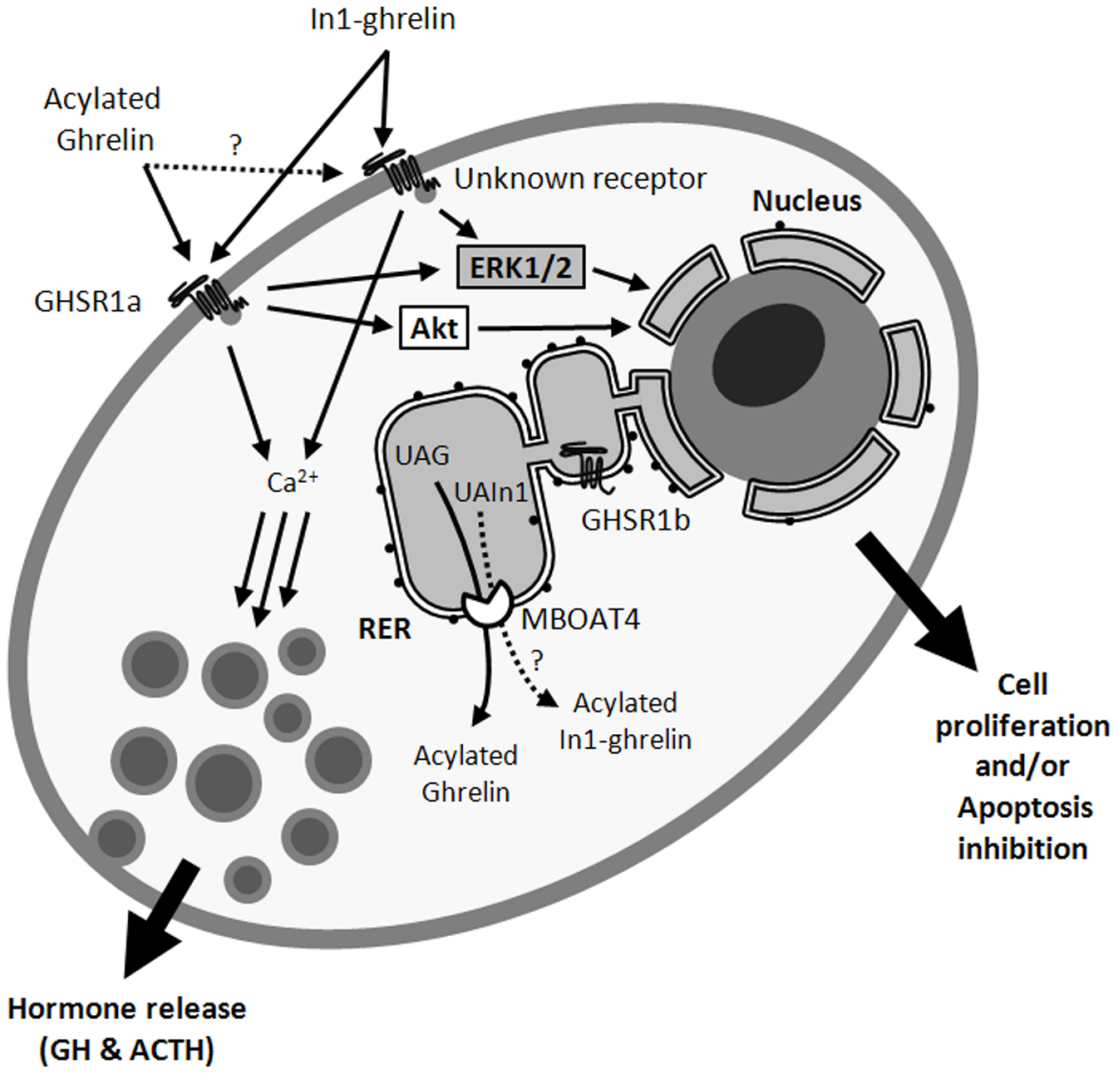
Working model summarizing the putative mechanisms and second messenger routes activated by ghrelin and In1-ghrelin in pituitary tumor cells. The data presented here analyzing different intracellular signaling pathways indicate that ghrelin and In1-ghrelin triggers ERK1/2 and Ca^2+^ mobilization, whereas only ghrelin activates Akt phosphorylation. We propose that an additional receptor besides GHSR1a may exist that would bind In1-ghrelin (and possibly acylated ghrelin) to evoke functional endpoints such as hormone release or cell proliferation. It contrast neither ghrelin nor In1-ghrelin would be able to act through GHSR1b. MBOAT4 (GOAT) is responsible for ghrelin acylation in Ser3, and as In1-ghrelin share this Ser3, it seems plausible that In1-ghrelin may be acylated as well.

Indeed, our study offers new clues in the understanding of the role of ghrelin system in the regulation of pituitary adenomas by demonstrating for the first time the significant overexpression of In1-ghrelin in all the pituitary adenoma subtypes analyzed compared to normal pituitaries. In fact, In1-ghrelin variant is the only component of the ghrelin system consistently overexpressed in pituitary adenomas, thus suggesting a putative utility of this variant for the development of new and more universal diagnostic, prognostic or therapeutic tools for the management of human pituitary adenomas. Indeed, In1-ghrelin is widely expressed in normal tissues but profoundly overexpressed in pathologic conditions such as breast cancer, wherein it can promote proliferation of breast cancer cell lines^6^. Here, we have demonstrated that the acylated In1-ghrelin could also play a relevant pathological role in pituitary adenomas. In particular, this study demonstrates that GH-oma cells respond to In1-ghrelin peptides by increasing [Ca^2+^]_i_, which was associated with augmented GH secretion *in vitro*^34^. Parallel administration of AG also increased [Ca^2+^]_i_ and promoted GH secretion by GH-oma cells, which is consistent with previous reports showing similar effects using ghrelin-analogs^35^. In addition, we demonstrate, for the first time, that treatment with In1-19 increased p-ERK1/2, but not p-Akt, levels in GH-omas. Interestingly, parallel administration with AG increased both, p-ERK1/2 as well as p-Akt, levels in GH-oma cells, which is also consistent with earlier findings in murine and human cell lines^36^,^37^,^38^ and, in primary pituitary cell cultures of a non-human primate model^39^. Although our results on intracellular signaling pathways ([Ca^2+^]_i_, as well as ERK and Akt activation) do not provide a definitive, complete elucidation of the intracellular signaling pathways involved in the response of In1-ghrelin peptide(s) and AG in GH-omas, we believe that the data presented provide novel evidence regarding how this novel ghrelin variant regulates some specific intracellular signaling pathways in GH-oma cells, and suggest the possibility that these pathways may be partially independent to those activated by AG in these cells. A working model summarizing the putative mechanisms and second messenger routes activated by ghrelin and In1-ghrelin in pituitary tumor cells is summarized in Figure 7.

Remarkably, In1-ghrelin also influenced other key, clinically relevant process, such as cell viability, in these tumors, since the In1-19 peptide significantly increased cell viability. Of note, this effect is more effective than that elicited by In1-40 peptide (p = 0.0079 at 72 h) or by AG itself (p = 0.0025 at 72 h), which modestly increased GH-oma cell viability, consistent with a previous study in rat pituitary somatotrope cell lines indicating that AG can stimulate cell proliferation^40^. In accordance, we found that In1-ghrelin overexpression also increased viability in GHoma cells, while In1-ghrelin silencing using a specific siRNA reduced cell viability, which, again, strongly suggests a relevant role of In1-ghrelin in GH-omas pathophysiology. Furthermore, we found that treatment with In1-19 also altered (decreased) apoptotic rate in GH-omas, whereas AG did not alter apoptotic rate as compared to vehicle-treated controls, which is in line with previous reports showing that AG treatment did not affect the apoptotic rate in other endocrine cell types (e.g. pancreatic cells)^38^. In fact, to the best of our knowledge, this is the first study examining the direct effect of AG or In1-ghrelin variant peptide on apoptotic rate on human GH-omas.

Overall, these results observed in response to In1-ghrelin peptide(s) treatment (i.e. an increase in proliferation rate and a decrease apoptotic rate) suggest that In1-ghrelin variant might represent a regulatory mechanism to control tumor growth in GH-oma cells. In line with this, it should be mentioned that these effects might be directly associated to the activation of MAPK signaling (i.e. ERK1/2-phosphorylation) observed in response to treatment with In1-ghrelin peptide, since the activation of this signaling pathway has been commonly associated to the regulation of cell growth and proliferation^41^.

In1-ghrelin peptides also increased [Ca^2+^]_i_ and stimulated ACTH release from human ACTH-oma cells and from the mouse AtT-20 corticotropinoma cell line. This was also observed with AG in human ACTH-omas, and is in line with previous data showing that AG is able to elicit an effect on ACTH release^42^, likely through an increase in [Ca^2+^]_i_^23^. In addition, In1-ghrelin overexpression also stimulated ACTH-oma cell viability while treatment with In1-ghrelin peptide In1-19, but not In1-40, revealed similar effects, suggesting that In1-19 peptide rather than In1-40 peptide could be likely responsible for the observed effects of In1-ghrelin overexpression in ACTH-producing derived cells. In accordance, we also found that treatment with mouse In2-ghrelin peptide increased cell viability in AtT-20 cell line which further supports the influence of this novel variant of the *GHRL* in the control of clinically relevant endpoints (i.e. cell viability and hormonal release) in pituitary adenomas of different species. Moreover, it should be mentioned that we had the opportunity to observe that In1-19 treatment was able to exert a clear inhibition in the apoptotic rate in one ACTH-oma sample included in the study which, together with the previous results observed in GH-omas and ACTH-omas, further support the notion that treatment with the In1-ghrelin peptides influences multiple, clinically relevant parameters on human pituitary adenomas, and may thus offer the possibility of identifying new therapeutic tools to treat these adenomas. Obviously, future studies, using larger number of human culture samples and additional, different types of assays are warranted to unequivocally establish if the overexpresion of In1-ghrelin variant observed in all the human pituitary adenomas analyzed herein is directly associated with an increase in proliferation and a decrease in apoptotic rate observed in these human pituitary pathologies.

In NFPAs, no functional studies had been implemented to date and, therefore, the role of the ghrelin system in this pathology remained unknown^1^. Accordingly, our study is the first to demonstrate that a subset of NFPA cells is responsive to AG and In1-ghrelin peptides in terms of [Ca^2+^]_i_ kinetics. More importantly, both AG and In1-ghrelin peptides (mainly In1-19) increased NFPA cells viability, which was further confirmed by In1-ghrelin overexpression experiments. As proof of concept, silencing of In1-ghrelin expression significantly reduced NFPA cell viability, which is particularly relevant in this kind of pituitary adenomas since the main comorbidities are derived from tumor overgrowth and mass effects^43^.

Results using [Ca^2+^]_i_ kinetic assays suggested the existence of a reduced responsiveness of PRL-omas to AG, in that only a low proportion of these pituitary adenoma cells altered their [Ca^2+^]_i_ in response to AG. Yet, this seems to be sufficient to elicit functional responses since AG treatment increases PRL secretion in this and in previous studies^35^. Conversely, In1-ghrelin peptides only elicited [Ca^2+^]_i_ responses in a negligible percentage of PRL-oma cells, which is in accordance with the lack of effect of these peptides in the modulation of PRL release. However, In1-40 but not In1-19 peptide was found to be as potent as AG in stimulating PRL-oma cell viability, which is in striking contrast with that observed in other pituitary adenoma subtypes wherein In1-19 was found to be more effective than In1-40 in all endpoints analyzed.

In sum, the data presented herein demonstrate, for the first time, a consistent upregulation of In1-ghrelin expression in the most predominant pituitary adenoma subtypes compared to normal pituitary. Moreover, the functional data collected strongly support the concept that In1-ghrelin substantially influences intracellular signaling, hormone secretion, and cell viability in pituitary adenoma cells. Hence, it seems reasonable to propose that the In1-ghrelin variant could play a relevant functional role in the regulation of pituitary adenoma pathology. As such, our results pave the way for using In1-ghrelin variant as a new tool to explore novel and more general diagnostic, prognostic and/or therapeutic targets in these human pathologies, where additional studies are warranted to determine the clinical implications of the alteration of specific components of the ghrelin family.

## Methods

### Patients and samples

Human pituitary specimens were obtained during transsphenoidal surgery from 169 patients [76 somatotropinomas, 57 non-functioning pituitary adenomas (NFPA), 29 corticotropinomas, and 7 prolactinomas]. In addition, 11 normal pituitaries (normal pituitary) pieces were included in the study [10 obtained from autopsies and one from a commercial source (pool of multiple individuals); CLONTECH; Palo Alto, CA, USA]. The methods were carried out in accordance with the approved guidelines, including the ethical standards of the Helsinki Declaration of the World Medical Association, University of Córdoba/IMIBIC and University Hospital Ethics Committees; additionally informed consent from each patient or relative, in case of autopsy, was obtained. All experimental protocols were approved by University of Cordoba/IMIBIC licensing committee. The phenotype of the pituitary samples collected (normal pituitaries or adenoma subtypes) was confirmed by three separate methods: examination by an anatomopathologist, testing the hormonal phenotype using single-cell secretion^23^ (not possible in the case of normal pituitaries), and molecular screening by quantitative real-time PCR (qPCR)^30^. Available demographic and clinical data are summarized in Table 1.

### Peptides

Human and rodent acylated ghrelin were commercially available (SC1357, and SC1356, respectively; PolyPeptide Laboratories, Limhamn, Sweden), while human, In1-ghrelin, and mouse, In2-ghrelin, acylated peptides were synthesized in collaboration with Ipsen Bioscience (Cambridge, MA, USA) and developed by CPC Scientific (Chinese Peptide Company, Hangzhou, China; see below). Although the mature endogenous In1/In2-ghrelin derived peptides have not yet been identified, pre-pro-In1-ghrelin and pre-pro-In2-ghrelin precursors exhibit target sites for protein-convertases suggesting a further proteolytic processing. As previously reported^6^, In1-ghrelin precursor processing could generate 19-aa or 40-aa long peptides (named In1-19: GSSFLSPEHQRVQVRPPHK and In1-40: GSSFLSPEHQRVQVRPPHKAPHVVPALPLSNQLCDLEQQR), which share with native ghrelin the initial 13-aa, including the acylation site at Ser3. On the other hand, we have previously reported^12^ that In2-ghrelin precursor processing could generate a single32-aa long peptide (named In2-ghrelin: GSSFLSPEHQKAQVSQSVSLSPHIYPDLCVCV), and similarly share with native mouse ghrelin the initial 13-aa.

The acylated In1-19, In1-40 and In2-ghrelin peptides were synthesized using manual solid-phase peptide synthesis starting with Fmoc-Lys(Boc)-Wang resin on a 0.5 mmol scale. The resins were treated with DCM/DMF (1:1) for 1 h, followed by standard Fmoc single coupling cycles with 1.5 mmol amino acid and coupling agent. All amino acids were Fmoc-protected except for Ser 3, which was unprotected. All amino acids were activated with HBTU or DICGly1 and HATU. Octanoic acid was coupled to Ser3 using 2 × 5 mmol octanoic acid and HOBt, followed by 2 × 10 mmol octanoic acid and HOBt. The peptides were then treated with a cocktail of TFA/EDT/Thioanisole/Phenol/H2O (87.5:2.5:5:2.5) for 2.5 h to remove the peptides from the resins. The peptides were confirmed by ESI MS and analytical RP-HPLC. The peptides were eluted with a gradient of Buffer B (0.09% TFA in 80% CH3CN/H2O) in aqueous 0.1% TFA. The peptides solubility was determined to be 1 mg/mL in water. Finally, peptide content was determined by AAA.

### Overexpression vectors and specific siRNAs

To perform overexpression experiments, ghrelin sequence was purchased, cloned in pCMV-Sport6-vector (Harvard Cancer Center, Boston, MA, USA) and was subcloned into the expression vector pCDNA3.1+ (Life Technologies, Grand Island, NY, USA). In1-ghrelin sequence was PCR-amplified (In1-Hind-Up: 5′-TCTCAAGCTTATGCCCTCCCCAGGGAC-3′ and In1-Bam-Low: 5′-TGTGGGATCCCTAGAGCTCGGGGCTGCAG-3′) and cloned into pCDNA3.1+. Empty pCDNA3.1+ (mock) was used in all the experiments as negative control. For silencing experiments, a battery of custom-designed siRNAs specifically targeted against the unique In1-ghrelin mRNA sequence was chemically synthesized by Life Technologies and their efficiency, specificity and appropriate concentration tested in human cell lines expressing In1-ghrelin (MDA-MB-231 breast cancer cell line and LnCAP prostate cancer cell line) (data not shown). Among them, the herein named In1-ghrelin siRNA (5′-GAGTCCTAAACAGACTGTT-3′) demonstrated a more robust silencing efficiency of In1-ghrelin, but not native ghrelin, in all the models tested (data not shown). As a negative control, a commercial, validated negative control (scramble) was used in all the experiments, which according to the manufacturer's instructions, has no significant sequence similarity to murine or human gene sequences (Silencer Select Negative Control No. 1 siRNA, Life Technologies, Green Island, NY, USA).

For transfection assays (overexpression or silencing), 200,000–500,000 cells were seeded on 6-well plates in serum containing medium during 24–36 h. Then, medium was replaced with antibiotic-antimycotic free medium. For overexpression experiments, 100 ng/50,000 cell of ghrelin or In1-ghrelin plasmid was diluted in 50 μL of Opti-MEM and, 0.5 μL of Lipofectamine 2000 (Life Technologies) were separately diluted in another 50 μL of Opti-MEM. Both solutions were incubated for 5 minutes at room temperature and then, were mixed and incubated for 30 minutes at room temperature. After this incubation period, the solution (total of 100 μL) was added to the cells/per well. For silencing experiments, 7.5 μL of Lipofectamine RNAiMAX (Life Technologies) was diluted in 100 μL of Opti-MEM and, 100 nM of In1-ghrelin siRNA was separately diluted in another 100 μL of Opti-MEM. Both solutions were incubated for 5 minutes at room temperature and then, were mixed and incubated for 30 minat room temperature. After this incubation period, the solution (total of 200 μL)was added to the cells/per well. In both cases (overexpression or silencing experiments), the solutions added to each well were incubated for 6–8 h at 37°C and then, medium was replacedwith serum containing medium for 18 h. Finally, cells were detached and seeded in 96-well plates for cell viability measurements and, some cells were collected for validation of the transfection efficiency (measure by qPCR).

### Cell lines and culturing

The mouse corticotroph pituitary derived cell line AtT-20/D16v-F2 (ATCC® CRL-1795) was cultured and maintained in Dulbecco's Modified Eagle's Medium (DMEM) complemented with 10% FBS, 100 U/ml penicillin/streptomycin, 0.024 M of 2-(4(2-hydroxyethyl)-1-piperazine)-ethane sulfonic acid (HEPES), and maintained at 37°C and 5% CO_2_, under sterile conditions. CHO-K1 cell line(ATCC® CCL-61) expressing recombinant human GHSR1a (GenBank accession number U60179) or GHSR1b (GenBank accession number U60181) were cultured and maintained in Ham's F12 media (Corning #10-080-CV) supplemented with 10% FBS, 2 mM L-Glutamine and 0.4 mg/mL Geneticin.

### RNA isolation, reverse-transcription, qPCR of human transcripts from normal pituitaries and tumor samples

RNA extraction, quantification, reverse-transcription as well as the development, validation and application of qPCR to measure the expression levels of different human transcripts have been previously reported elsewhere by our group^23^,^44^,^45^. Due to asynchronically collection of samples, total RNA was extracted from tumoral pieces with two commercial kits following the manufacturer's protocol: Absolutely RNA RT-PCR Miniprep Kit (Agilent, La Jolla, CA, USA) with deoxyribonuclease treatment and AllPrep DNA/RNA/Protein Mini Kit followed by deoxyribonuclease treatment using RNase-Free DNase Set (Qiagen, Limburg, Netherlands). AtT-20 cell cultures were processed to isolate total RNA using TRIzol Reagent (Life Technologies, Barcelona, Spain) following the manufacturer's protocol and subsequently treated with DNase (Promega, Barcelona, Spain). In all cases, total RNA concentration and purity was assessed using Nanodrop 2000 spectrophotometer (Thermo Scientific, Wilmington, NC, USA), and subsequently retro-transcribed using random hexamer primers and the cDNA First Strand Synthesis kit (MRI Fermentas, Hanover, MD, USA). cDNA derived from pituitary tissue and AtT-20 cell line were amplified by qPCR, where samples were run, in the same plate, against a standard curve to estimate mRNA copy number (1, 10^1^, 10^2^, 10^3^, 10^4^, 10^5^, and 10^6^ copies of synthetic cDNA template for each transcript) and a No-RT sample as a negative control. qPCR was performed using Brilliant II or III SYBR Green Master Mix in the Stratagene Mx3000p instrument (Agilent, La Jolla, CA, USA) as previously described^23^,^44^,^45^. Thermal profile consisted of a initial step at 95°C for 10 minutes, followed by 40 cycles of denaturation (95°C for 30 seconds), annealing (61°C for 1 minute), and extension (72°C for 30 seconds); and finally, a dissociation cycle to verify that only one product was amplified. In human pituitary samples, expression levels of native ghrelin, In1-ghrelin variant, GHSR1a, GHSR1b, MBOAT4 and ACTB were determined, when possible, while in mouse AtT-20 cell line expression levels of native ghrelin, In2-ghrelin variant, Ghsr, Mboat4 and Actb, Ppia and Hprt were determined. Specific sets of primers used in this study are shown in Supplemental Table 1. To control for variations in the amount of RNA used and the efficiency of the reverse-transcription reaction and, the expression level (copy-number) of each transcript was adjusted by ACTB expression in human samples, and by a normalization factor calculated with the expression levels of Actb, Ppia and Hprt using GeNorm 3.3^46^ in mouse AtT-20 cell line. It should be noted that, as previously reported^44^,^45^ and based on the stringent criteria to maximize specificity and efficiency, the qPCR technique, as applied, can be used to accurately quantify copy numbers for all human transcripts included in this study.

### Primary pituitary cell culture

Pituitary adenoma specimens were dispersed into single cells by enzymatic and mechanical disruption and cultured onto tissue culture plates in serum containing medium, as previously described^23^,^30^,^47^,^48^. Briefly, pituitary samples were resected by endoscopic transsphenoidal surgery and transferred to sterile cold (4°C) culture medium (S-MEM, Gibco, Madrid, Spain) supplemented with 0.1% BSA, 0.01% L-glutamine, 1% antibiotic-antimycotic solution, and 2.5% HEPES. Samples were minced under sterile conditions into 1–2 mm^3^ pieces. Some pieces were stored at −80°C for posterior RNA isolation and, when possible, the remaining tissue samples were washed and incubated in 30 mL S-MEM medium supplemented 0.3% trypsin (Beckson, Dickinson and Company, Sparks, MD, USA) in a spinner flask (Bellco Glass, Vineland, NJ, USA) for 2 h of gentle shaking at 37°C and then, incubation was continued for another 5 min in presence of 1 mg of DNAse I (Roche, Mannheim, Germany). Dispersed cells were decanted by centrifugation and then, by repeated aspiration into a smoothtipped glass Pasteur pipette. Cells suspensions were washed once in 4.5 g/L glucose containing DMEM medium (Gibco, Madrid, Spain) supplemented with 0.1% BSA, 0.01% L-glutamine, 1% antibiotic-antimycotic solution, and 2.5% HEPES. Cell number and viability was estimated by the Trypan blue exclusion test in Neubauer chamber, where viability was always 90–100%.

### Analysis of hormone secretion

To examine the effects of ghrelin system components (4- and/or 24 h-incubation) on pituitary hormone release from primary pituitary adenoma and/or AtT-20 cell line, 100,000–200,000 cell/well were used. As previously reported^15^, hormone concentrations were measured in the culture media derived from human samples according to the pituitary adenoma type using commercial ELISAs [growth hormone, GH; adrenocorticotropin, ACTH; and prolactin, PRL (reference numbers: EIA-3552, EIA-3647 and EIA-1291, respectively; DRG, Mountainside, NJ, USA)] following the manufacturer's instructions. All the information regarding specificity, detectability, and reproducibility for each of the assays can be accessed at the web site of the company.

### Measurement of free cytosolic calcium mobilization ([Ca^2+^]_i_)

As previously reported, changes in [Ca^2+^]_i_ in single cells were measured using fura-2AM (50,000 cells/coverslip; Molecular Probes, Eugene, OR)^23^,^30^. Briefly, primary pituitary adenoma cells were grown onto glass cover slips for 36–48 h (35-mm plates), and then incubated for 30 min at 37°C with fura-2AM in phenol red-free DMEM containing 20 mM NaHCO_3_ (pH 7.4). Coverslips were washed with phenol red-free DMEM and set on a Sykes-Moore chamber (Bellco Glass, Madrid, Spain) and placed on an inverted microscope Eclipse TE2000-E (Nikon, Tokyo, Japan) coupled to a digital camera ORCA II BT (Hamamatsu Photonics, Hamamatsu, Japan). Cells were examined for changes in [Ca^2+^]_i_ after the appropriate treatment (native ghrelin, In1-19 or In1-40) using a 40× objective with Immersion Oil Type NF (Nikon, Tokyo, Japan) while exposed to alternating 340–380 nm light beams, and the intensity of light emission at 505 nm was measured every 5 seconds using MetaFluor Software (Imaging Corp., West Chester, PA, USA). Phenol red-free DMEM and ionomycin (Sigma-Aldrich, Madrid, Spain) were used as negative and positive control, respectively. Changes in [Ca^2+^]_i_ in CHO-K1 cell line expressing recombinant GHSR1a or GHSR1b receptor were measured using Calcium 4 Explorer Kit (Molecular Devices, Sunnyvale, CA, USA). Briefly, cells were incubated at 37°C, 5% CO_2_ overnight in growth media. After 24 hours, growth media was replaced with Calcium 4 indicating dye in HBSS buffer containing 20 mM HEPES and 0.1% BSA and incubated for 1 hour at 37°C. Fluorescent signals were then monitored using the FLIPR Tetra (Molecular Devices, Sunnyvale, CA, USA) during which cells were stimulated with compounds. Dose-response curves were analyzed using GraphPad Prism v5.04 (La Jolla, CA, USA).

### Measurement of ERK1/2 and Akt signaling pathways by western blotting

500,000 cells were cultured in 6-well plates and incubated for 8 minutes with acylated ghrelin, acylated In-19 and vehicle-treated controls. Briefly, after the corresponding treatment, medium was removed and cells were washed twice using PBS, detached using a scrapper and immediately lysed in pre-warmed SDS-DTT sample buffer at 65°C (62,5 mM Tris-HCl, 2% SDS, 20% glycerol, 100 mM DTT and 0,005% bromophenol blue) followed by sonication for 10 seconds and boiling for 5 minutes at 95° C as previously described^49^,^50^. Proteins were separated by SDS-PAGE and transferred to nitrocellulose membranes (Millipore, Darmstadt, Germany), and then membranes were blocked with 5% non fat dry milk in Tris-buffered saline/0.05% Tween 20 and incubated with the primary antibodies for ERK1/2, p-ERK1/2, p-Akt, Akt and the appropriate secondary antibodies [primary total ERKs (Santa Cruz, CA, USA); primary p-ERK1/2, p-Akt, and Akt as well as, secondary anti-rabbit antibody from Cell Signaling (Danvers, MA, USA)]. Proteins were developed using an enhanced chemiluminescence detection system (GE Healthcare, UK) with dyed molecular weight markers. A densitometric analysis of the bands was carried out with ImageJ software^51^. Relative phosphorylation of ERK and Akt was obtained from normalization of p-ERK1/2 or p-Akt against the total ERK1/2 or β-Akt, respectively.

### Measurements of cell viability

As previously reported, cell viability was estimated using alamar-blue reagent (10,000 cells/well-plate; Biosource International, Camarillo, CA, USA)^30^,^50^,^52^. Briefly, cells were serum-starved for 12–16 h before the measurements and treated with serum-free medium containing 10% alamar-blue for 3 h. Reduction of alamar-blue reagent was quantified exciting at 560 nm and reading at 590 nm using the FlexStation III system (Molecular Devices, Sunnyvale, CA, USA). Subsequently, media was replaced by serum-containing alamar-blue free medium and incubated for additional 24–72 h, measuring the alamar-blue reduction every 24 h. In experiments using the AtT-20 cell line, IGF1 and Paclitaxel (Sigma-Aldrich, Madrid, Spain) were used as positive and negative controls, respectively.

### Measurements of apoptosis

To evaluate the apoptotic rate in GH-oma and ACTH-oma cells, 150,000 cells/well were plated and cultured for 36–48 h. Then, cell cultures were incubated for 12 h with AG, acylated In1-19, hydrogen peroxide (used as positive control) and vehicle-treated controls. After the incubation period, culture cells were processed as follows: step-1) media were collected, centrifuged 5 min at 1,200 rpm and, the supernatant was discarded and the pellet was maintained; step-2) cells were washed with PBS, detached using a cell scrapper and collected together with the previous pellet (step-1). Then, the mixture was centrifuged 5 min at 1,200 rpm and the supernatant was discarded while the pellet was processed following manufacturer's instructions of Annexin-V-FITC/propidium iodide staining assay (Bender Medsystems, Barcelona, Spain) and measurement were carried out by flow cytometry (Beckman Coulter, Coulter Epics XL, Madrid, Spain).

### Statistical analysis

Data were evaluated for heterogeneity of variance using the Kolmogorov–Smirnov test. Statistical differences were assessed by Mann–Whitney U test or by one-way or two-way ANOVA followed by Fisher's test. Correlations were studied using Spearman's correlation test. Data are expressed as median ± interquartile ranges or as mean ± S.E.M. p < 0.05 was considered significant. All statistical analyses were performed using the GraphPad Prism5 (La Jolla, CA, USA) or the SPSS software (IBM, New York, NY, USA).

## Supplementary Material

Supplementary Information Supplemental Data

## Figures and Tables

**Table 1:** Demographic and clinical parameters of patients included in this study

	GH-omas	ACTH-omas	NFPAs	PRL-omas
Number of cases	76	29	57	7
Mean age (years)	42.7	35.9	56.5	32.7
Gender (% of women)	51%	75%	39.6%	42.8%
Tumor size (% macroadenomas)	92%	36%	100%	100%
Extrasellar growth	84%	60%	87%	50%

**Table 2:** Results from free cytosolic calcium kinetics assays in tumoral pituitary cells in response to ghrelin gene derived peptides

**GH-omas**							
	# samples	Cells analyzed	% cell resp.	% Max.	Error	T resp.	Error
*AG*	27/27	1500	74.0	199.4	9.7	18.9	2.2
*In1-19*	13/15	983	60.4	179.3	12.6	15.9	1.4
*In1-40*	9/13	520	49.2	142.7	6.4	17.6	3.1
**ACTH-omas**							
	# samples	Cells analyzed	% cell resp.	% Max.	Error	T resp.	Error
*AG*	9/9	742	74.1	217.6	19.0	14.3	6.6
*In1-19*	3/5	230	43.9	187.5	35.2	20.0	8.9
*In1-40*	3/4	143	37.8	161.1	8.0	25.0	1.3
**NFPA**							
	# samples	Cells analyzed	% cell resp.	% Max.	Error	T resp.	Error
*AG*	9/15	408	29.4	162.0	9.1	23.6	7.9
*In1-19*	7/12	367	18.0	152.8	10.9	14.0	3.0
*In1-40*	1/6	29	10.3	118.7	2.3	55.0	8.5
**PRL-omas**							
	# samples	Cells analyzed	% cell resp.	% Max.	Error	T resp.	Error
*AG*	2/6	217	19.8	178.5	1.4	35.1	7.9
*In1-19*	1/5	160	4.4	155.3	7.5	18.6	5.0
*In1-40*	1/3	88	8.0	147.5	8.8	29.3	4.8
