# Supplementary material for: In1-ghrelin splicing variant is overexpressed in pituitary adenomas and increases their aggressive features
Source: Sci Rep. 2015 Mar 4;5:8714. doi: 10.1038/srep08714 (PMC4649711; doi:10.1038/srep08714)
Supplement: Supplementary Information — Supplemental Data [file srep08714-s1.pdf]

In1-ghrelin splicing variant is overexpressed in pituitary adenomas and increases their aggressive features

Alejandro Ibáñez-Costa[1], Manuel D. Gahete[1], Esther Rivero-Cortés[1], David Rincón-Fernández[1], Richard Nelson[2], Manuel Beltrán[3], Andrés de la Riva[4], Miguel A. Japón[5], Eva Venegas-Moreno[6], Mª Ángeles Gálvez[7], Juan A. García-Arnés[8], Alfonso Soto-Moreno[6], Jennifer Morgan[2], Natia Tsomaia[2], Michael D. Culler[2], Carlos Dieguez[9], Justo P. Castaño[1]*, Raúl M. Luque[1]*

Supplemental Table 1. Primers used for quantification of human and mouse transcripts

Transcript	Accession number	Sense	Antisense	Size bp
		**Human**		
Ghrelin	NM_016362.3	CACCAGAGAGTCCAGCAGAGA	CCGGACTTCCAGTTCATC	132
In1-ghrelin	GU942497.1	TCTGGGCTTCAGTCTTCTCC	GTTCATCCTCTGCCCCTTCT	215
MBOAT4	NM_001100916.1	TTGCTCTTTTTCCCTGCTCTC	ACTGCCACGTTTAGGCATTCT	161
GHSR1a	NM_198407.2	TGAAAATGCTGGCTGTAGTGG	AGGACAAAGGACACGAGGTTG	148
GHSR1b	NM_004122.2	GGACCAGAACCACAAGCAAA	AGAGAGAAGGGAGAAGGCACA	107
ACTB	NM_001101.3	ACTCTTCCAGCCTTCCTTCCT	CAGTGATCTCCTTCTGCATCCT	176
		**Mouse**		
Ghrelin	NM_021488.4	TCCAAGAAGCCACCAGCTAA	AACATCGAAGGGAGCATTGA	126
In2-ghrelin	DO_993169	GCTGTCTTCAGGCACCATCT	GTGGCTTCTTGGATTCCTTTC	226
Mboat4	NM_001126314.2	ATTTGTGAAGGGAAGGTGGAG	CAGGAGAGCAGGGAAAAAGAG	120
Ghsr	NM_177330.3	TCAGGGACCAGAACCACAAA	CCAGCAGAGGATGAAAGCAA	71
Actb	M12481	CTGGGACGACATGGAGAAGA	ACCAGAGGCATACAGGGACA	205
Ppia	NM_008907	TGGTCTTTGGGAAGGTGAAAG	TGTCCACAGTCGGAAATGGT	109
Hprt	NM_013556	CAGTCAACGGGGGACATAAA	AGAGGTCCTTTTCACCAGCAA	183

Supplemental Table 2. Significant correlations between expression levels of ghrelin system components in pituitary adenomas[1]

		Ghrelin	In1-ghrelin	MBOAT4	GHSR1a	GHSR1b
GH-omas	Ghrelin		0.512 ***	0.493***		
	In1-ghrelin	0.512 ***		0.322 *		
	MBOAT4	0.493***	0.322 *			
	GHSR1a					0.849 ***
	GHSR1b				0.849 ***	
ACTH-omas	Ghrelin					
	In1-ghrelin			0.510 *	0.560 *	
	MBOAT4		0.510 *			
	GHSR1a			0.560 *		
	GHSR1b					
NFPAs	Ghrelin		0.343*	0.564 ***	0.375 **	
	In1-ghrelin	0.343*		0.452 **	0.352 *	0.436 **
	MBOAT4	0.564 ***	0.452 **		0.601 ***	0.522 ***
	GHSR1a	0.375 **	0.352 *	0.601 ***		
	GHSR1b		0.436 **	0.522 ***		

[1]Only significant correlations are shown in the table

Supplemental Table 3. Half-maximal Calcium signaling activation (EC_{50}) in response to *GHRL*-gene derived peptides

	GHSR1a-transfected cells	GHSR1b-transfected cells
Acylated		
Ghrelin	1.5 ± 0.4	>3000
In1-19	2.8 ± 1.5	>3000
In1-40	17.2 ± 2.2	>3000

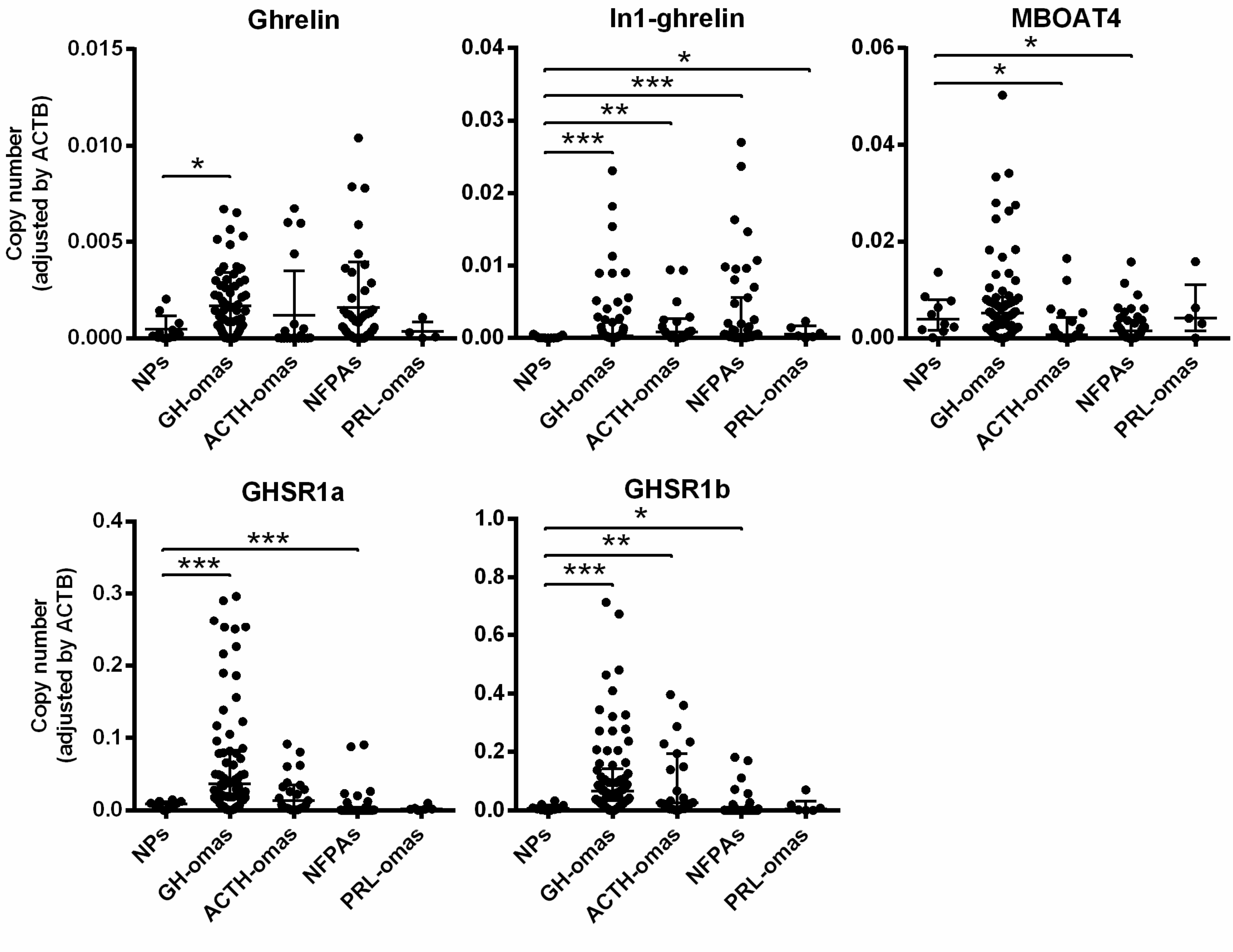

Supplemental Figure 1. Expression profile of ghrelin system components in normal and tumoral pituitaries. The expression of ghrelin system components (ghrelin, In1-ghrelin variant, MBOAT4, GHSR1a and GHSR1b) was determined by qPCR in a battery of 169 pituitary adenomas (including 75 GHomas, 29 ACTHomas, 57 NFPAs and 7 PRLomas) and compared to the expression levels found in 11 normal pituitaries (NPs). Data represent median ± interquartile range of absolute expression levels (copy number) of each transcript adjusted by the expression levels of a control gene (ACTB). Asterisks (*, $p<0.05$; **, $p<0.01$ and ***, $p<0.001$) indicate data that differ from NP expression by Mann-Whitney U test.
